# Genome Sequence of the Butyrate*-*Producing Anaerobic Bacterium Anaerostipes hadrus PEL 85

**DOI:** 10.1128/genomeA.00224-15

**Published:** 2015-04-02

**Authors:** Ravi Kant, Pia Rasinkangas, Reetta Satokari, Taija E. Pietilä, Airi Palva

**Affiliations:** Department of Veterinary Biosciences, Faculty of Veterinary Medicine, University of Helsinki, Helsinki, Finland

## Abstract

Anaerostipes hadrus PEL 85, which was isolated from human feces, is a Gram-positive rod-shaped bacterium. The species may play an important role in gut health, as it was previously reported to produce butyric acid. Here, we present the genome assembly of PEL 85, a novel strain of A. hadrus.

## GENOME ANNOUNCEMENT

Recently, Eubacterium hadrum, isolated in 1976 by Moore, Johnson, and Holdeman (1), was reclassified as Anaerostipes hadrus (2). A. hadrus has been estimated to represent >2% of the total microbiota in a healthy human colon (3), and the species has been shown to produce butyrate (2). Butyrate has been demonstrated to have a positive impact on gastrointestinal tract homeostasis, as it promotes the growth of intestinal epithelial cells, increases the expression of tight junction proteins, and acts as an anti-inflammatory agent (4–8). Hence, butyrate-producing bacteria are generally thought to be health promoting.

A. hadrus-like bacteria were found to be more abundant in the feces of healthy controls in our study concerning diarrhea-predominant irritable bowel syndrome (IBS-D) (9). The study was based on high-throughput 16S rRNA gene sequencing, and the health-associated phylotype was referred to as Ruminococcus torques phylotype 93%. A. hadrus-like bacteria were present also in IBS subjects but in significantly lower amounts than those in healthy controls (9). We isolated a strain belonging to the same phylotype from the feces of a 31-year-old Finnish female while charactering microbiota in intestinal disorders. The woman had a diagnosis of celiac disease, maintained a gluten-free diet, and suffered from IBS-D-like symptoms. The fecal sample was cultivated anaerobically in a dilution series on reinforced clostridial medium (RCM) agar. The obtained colonies were screened by a quantitative PCR assay specific to the phylotype R. torques 93% (9), and a pure culture of the isolate PEL 85 was achieved by using sequential cultivations. A. hadrus PEL 85 is a Gram-positive rod-shaped anaerobic bacterium.

The genomic DNA was extracted using the Wizard genomic DNA purification kit (Promega), and the entire genome of A. hadrus PEL 85 was sequenced using the Roche 454 Life Sciences GS FLX system. The obtained sequences were assembled using Newbler, resulting in 23× coverage of the genome.

Protein-coding sequences (CDSs) were predicted using a combination of GeneMark and Glimmer3 (10, 11), followed by manual curation of the start sites. The remaining intergenic regions were reanalyzed for missed CDSs using BLASTx (12). Annotation transfer was performed based on the BLASTp search, followed by Rapid Annotations using Subsystems Technology (RAST) analysis (13) and manual verification.

The unclosed draft genome of A. hadrus PEL 85 contains 3,542,561 nucleotides. The overall G+C content of the chromosome is 36.6%. The chromosome contains 3,543 protein-coding sequences (CDSs), 58 tRNA genes, and 3 rRNAs. Putative functions were predicted for 2,111 CDSs (60%), and 1,432 (40%) protein-coding genes were found to be hypothetical or have an unknown function. Surprisingly, a very low percentage of CDSs with predicted signal peptides (144 [4.1%]) may indicate limited interactions of A. hadrus PEL 85 with its environment. It is noteworthy to mention that the genome of A. hadrus PEL 85 contains >45 putative butyrate-associated genes, including 9 butyryl-coenzyme A (CoA) dehydrogenase-linked genes having a major role in butyrate production.

### Nucleotide sequence accession number.

The draft genome sequence of A. hadrus PEL 85 is available in GenBank under the accession no. JYFK00000000.
